# Avoiding inappropriate urinary catheter use and catheter-associated urinary tract infection (CAUTI): a pre-post control intervention study

**DOI:** 10.1186/s12913-017-2268-2

**Published:** 2017-05-02

**Authors:** Vicki Parker, Michelle Giles, Laura Graham, Belinda Suthers, Wendy Watts, Tony O’Brien, Andrew Searles

**Affiliations:** 1 School of Nursing, University of New England, Armidale, NSW Australia 2351; 2 Hunter New England Nursing and Midwifery Research Centre, James Fletcher Campus, Gate Cottage, 72 Watt St, Newcastle, NSW Australia 2300; 3 Respiratory and General Medicine, John Hunter Hospital, Locked Bag 1 HRMC, New Lambton Heights, NSW Australia 2310; 4 School of Nursing and Midwifery, University of Newcastle, Callaghan, NSW Australia 2308; 5 Hunter Medical Research Institute (HMRI), New Lambton Heights, NSW Australia 2305

**Keywords:** Healthcare-associated infection, Catheter-associated urinary tract infection, Multifaceted intervention, Evidence-based practice

## Abstract

**Background:**

Urinary tract infection (UTI) as the most common healthcare-associated infection accounts for up to 36% of all healthcare-associated infections. Catheter-associated urinary tract infection (CAUTI) accounts for up to 80% of these. In many instances indwelling urinary catheter (IDC) insertions may be unjustified or inappropriate, creating potentially avoidable and significant patient distress, embarrassment, discomfort, pain and activity restrictions, together with substantial care burden, costs and hospitalisation. Multifaceted interventions combining best practice guidelines with staff engagement, education and monitoring have been shown to be more effective in bringing about practice change than those that focus on a single intervention. This study builds on a nurse-led initiative that identified that significant benefits could be achieved through a systematic approach to implementation of evidence-based practice.

**Methods:**

The primary aim of the study is to reduce IDC usage rates by reducing inappropriate urinary catheterisation and duration of catheterisation. The study will employ a multiple pre-post control intervention design using a phased mixed method approach. A multifaceted intervention will be implemented and evaluated in four acute care hospitals in NSW, Australia. The study design is novel and strengthened by a phased approach across sites which allows for a built-in control mechanism and also reduces secular effects. Feedback of point prevalence data will be utilised to engage staff and improve compliance. Ward-based champions will help to steward the change and maintain focus.

**Discussion:**

This study will improve patient safety through implementation and robust evaluation of clinical practice and practice change. It is anticipated that it will contribute to a significant improvement in patient experiences and health care outcomes. The provision of baseline data will provide a platform from which to ensure ongoing improvement and normalisation of best practice. This study will add to the evidence base through enhancing understanding of interventions to reduce CAUTI and provides a prototype for other studies focussed on reduction of hospital acquired harms. Study findings will inform undergraduate and continuing education for health professionals.

**Trial registration:**

ACTRN12617000090314. Registered 17 January 2017. Retrospectively registered.

**Electronic supplementary material:**

The online version of this article (doi:10.1186/s12913-017-2268-2) contains supplementary material, which is available to authorized users.

## Background

Urinary tract infection (UTI) is considered the most common healthcare-associated infection (HAI) [1], accounting for up to 36% of all healthcare-associated infections (HAIs) [2]. Catheter-associated urinary tract infections (CAUTIs) represent the majority of UTIs (up to 67% of UTIs in all hospital inpatients [3], and up to 97% in ICUs [4]). Between 12 and 16% of hospitalised patients may receive a short term indwelling urinary catheter (IDC) [5], and many of these IDC insertions have been identified as unjustified or inappropriate [6]. CAUTI risk increases considerably with duration of catheterisation [7], and generates substantial care burden and significant hospitalisation costs, patient distress, embarrassment, discomfort, pain and activity restrictions [7–9]. A recent Australian study indicated that 1.7% of inpatients, hospitalised for > 48 h, contract a UTI, adding additional days (mean = 4) to their length of stay (LoS) [10].

CAUTI is possibly the most preventable HAI [11], with significant potential cost savings. According to Mitchell et al.’s (2016) [10] calculations, there are ~380,600 extra public hospital bed-days used each year in Australia due to healthcare-associated urinary tract infections (the majority being catheter-associated [3]). Umscheid et al. (2011) [11] estimate that each CAUTI costs between $1200 and $4700 USD. In the Australian setting, Jackson et al. (2011) estimated that the costs associated with a patient diagnosed with CAUTI are twice as much as a patient not affected by CAUTI [12].

### Preventing CAUTI

Worldwide, there has been renewed interest and research into reducing the incidence of CAUTI, especially in the USA, with the introduction of non-payment for ‘reasonably preventable’ hospital-acquired complications [13]. An integrative review by Meddings et al. (2014) [1] evaluated interventions up to October 2012 to reduce IDC usage and CAUTIs. Meddings et al. (2014) found that interventions to reduce inappropriate IDC use, and bundles of interventions focusing on reducing unnecessary catheter use and general infection control were successful in reducing catheter use [1]. A component common to most urinary catheter bundles is timely catheter removal [14–20]. Meddings et al. recognised the importance of addressing socioadaptive factors in successfully implementing interventions [1]. These socioadaptive factors have since been addressed in the USA with the Agency for Healthcare Research and Quality (AHRQ) Comprehensive Unit-based Safety Program (CUSP). In a national US study, Saint et al. implemented CUSP in 926 units, and found a significant reduction in catheter use and CAUTI in non-ICUs [19]. Clinician education about recommended practice is a key part of interventions to address catheter use and CAUTI; nine studies since the Meddings et al. 2014 integrative review implemented a hospital (or multi-hospital) intervention to reduce CAUTI, and all included some form of education [14, 17, 19, 21–27]. Indeed, a systematic review of interventions to reduce device-related infections found that all interventions had some form of education as a key component [28].

From evaluating the literature on hospital-wide and multi-hospital interventions designed to reduce urinary catheter use and CAUTI, a gap was identified in study design; all identified studies used a pre-post design, which does not account for secular trends.

Studies investigating use of IDCs and CAUTI have been lacking in the Australian context [29, 30]. Extant literature includes hospital-based rates of IDC usage and/or rates of CAUTI, with some discussion of documentation, appropriate indications for IDC, and staff knowledge. Wynne et al. (2014) [29] found a point prevalence of 12.4% of patients with IDC in situ, in a tertiary teaching hospital in Melbourne. This included an acute inpatient facility and a sub-acute aged care and rehabilitation service. Wynne et al.’s study did not report on the days IDC in situ or prevalence of CAUTI, and a differentiation between short-term and long-term IDC usage was not made. So et al. (2014) conducted a chart audit in a hospital in Sydney, finding catheter utilisation of 11%. A study of staff and patient knowledge of IDC usage in two general medical wards in a Melbourne hospital found that the mean time an IDC was in situ was 5.8 days, and that a physician’s awareness of IDC presence was significantly associated with a shorter time IDC in situ [31]. Giles et al. (2015), in a pilot study, found the prevalence of IDCs in two wards in an Australian hospital (urology ward = 25%; orthopaedic ward = 31%), and rate of CAUTI = 2.2% [32]. Giles et al. then went on to describe the development and pilot of a bundled approach to target IDC utilization and CAUTI, however results of the intervention were not reported. A large point prevalence study in six Australian hospitals by Gardner et al. (2014) found a CAUTI prevalence of 0.9%, and urinary catheter prevalence of 26.3% (88.7% of these being IDCs) [33].

The foregoing studies highlight a gap in knowledge in the Australian healthcare context, in that there have been no studies investigating the effects of an intervention on reducing IDC utilisation and CAUTI rates in an acute care setting.

A multifaceted evidence-based intervention was piloted in two wards in an acute care hospital in the Hunter New England Local Health District, leading to a 50% reduction in IDC insertions, significantly reduced IDC duration and number of patients treated for CAUTI [34].

Building from the positive results from the aforementioned pilot, the present study aims to implement and evaluate an intervention across four acute care hospitals in NSW, Australia. To control for secular trends, implementation of the intervention will be phased across the hospitals.

## Methods/Design

### Aims

The primary aim of the study is to reduce IDC usage rates by reducing inappropriate urinary catheterisation and duration of catheterisation.

The secondary aims of the study are to identify:

1. The current inpatient indwelling urinary catheter usage rate and incidence of CAUTI;
2. If the implementation and adherence to bundled catheter care (BCC) reduces IDC use and CAUTI;
3. How effective BCC is in improving IDC-related outcomes;
4. The barriers and enablers to successful implementation of BCC; and
5. The cost benefits of implementation.

### Design

**Fig. 1:**
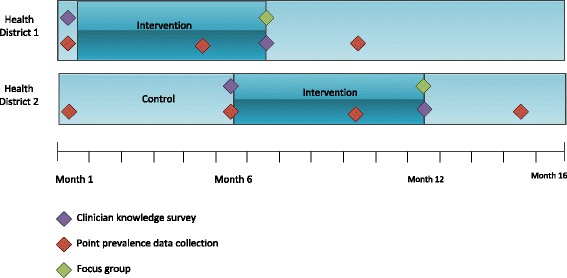
Study Design. Data collection points are indicated with diamonds on timeline

The study will employ a multiple pre-post control intervention design using a phased mixed method approach (Fig. 1).

Implementation of the intervention across four acute care hospitals will be staged, with multiple clusters in each of two implementation stages. Pre and post point prevalence data comparison will occur within all hospitals pre and post intervention, as well as between the two Health Districts as detailed in Fig. 1.

The staged implementation of the intervention allows for a control between the two Health Districts.

A mixed method design provides a platform to explore in-depth existing barriers and enablers related to implementing practice change. The sequential phased nature of the study ensures that the necessary evidence is available to inform the subsequent implementation phase of the study. The focus groups will identify barriers and enablers to implementation and uptake and will inform strategies to embed the intervention into normal practice. Questions in the focus groups will be informed by results from the point prevalence and clinician surveys.

The control will be usual urinary catheterisation practice, i.e., no intervention or implementation strategies. NSW Health evidence-based practice guidelines for “Adult Urethral Catheterisation for Acute Care Settings” [35], and local clinical practice guidelines for urinary catheterisation for each Health District are available for all clinicians, and can be accessed online.

The Clinical Excellence Commission (CEC), a corporation addressing patient safety and clinical quality in the NSW Health context, established a CAUTI project in 2014 to “help healthcare professionals in reducing the incidence of CAUTIs in acute care settings” [36]. A urinary catheterisation course on an online NSW Health learning platform also exists [37].

### Setting

The intervention sites are four acute care hospitals from two Health Districts in NSW, Australia. Hospitals have been purposively selected, matched on total bed numbers, activity type and activity levels (See Table 1).

**Table 1:** Setting

Health District	Facility	Beds
1	Hospital A	360
	Hospital B	260
	Total beds Health District 1 = 620	
2	Hospital C	549
	Hospital D	318
	Total beds Health District 2 = 867	

A key difference between the two health districts is the system used for medical health records: Health District 1 uses paper-based medical records, whereas Health District 2 uses electronic medical records. Data collection methods and training have been individualised to accommodate these differences.

### Data collection

Three main types of data will be collected:

- Pre and post implementation point prevalence and patient demographics (quantitative)
- Pre and post implementation clinician knowledge and competence (quantitative)
- Post implementation perceived barriers and enablers to implementation (qualitative)

Data collection types and details are outlined in Table 2, and the data collection timepoints are displayed in Fig. 1.

**Table 2:** Data collection sources and methods

Data	Data collection method	Data source(s)	Data collected	Data collection timepoint(s)
IDC usage rate and incidence of CAUTI	Online data collection tool	- Patient medical records – facility-wide across all four hospitals - Bedside observation - Infection control database	- Urinary catheter presence - Days catheter in situ - CAUTI rate	- Baseline - 4 months post-implementation commencement - 9 months post-implementation commencement
Patient profile	Data extraction and then merge with data from point prevalence	- Electronic patient management systems	- Patient demographics including age, gender, weight, diagnosis, type of admission	- Baseline - 4 months post-implementation commencement - 9 months post-implementation commencement
Clinician knowledge and competency	Online survey	- Clinicians (all nurses and medical officers invited from participating hospitals)	- Clinician competency - Clinician knowledge of CAUTI prevention - Perception of unit-based culture	- Baseline - 6 months post-implementation commencement
Barriers and enablers to implementation	Focus group	- Clinicians (6–8 per facility) (all nurses and medical officers invited from participating hospitals)	- Perceived barriers and enablers to implementation	- 6 months post-implementation commencement

The point prevalence data will be collected by project staff (clinical nurse consultants, research assistant), nurse and midwife clinicians and clinical nurse educators from each hospital. Training will be administered to all clinicians involved on data collection techniques and definitions prior to collection, and they will be paired where possible with members of the research team. Data collection staff will go to every inpatient bed on every adult inpatient ward in the hospital and input data into a firewall protected online survey tool. Survey data will then be exported and merged with other electronically extracted demographic patient data into statistical package STATA [38] for analysis.

### Exclusion and inclusion criteria

Point prevalence data will be collected from all adult inpatient wards across four hospitals in two Health Districts (excluding emergency departments, operating theatres and day only wards).

### Multifaceted intervention

The intervention will be delivered in all adult inpatient wards, emergency departments, and operating theatres in all four hospitals. The key component of the intervention is the evidence-based “No CAUTI” bundle (Table 3). To support implementation of the No CAUTI bundle, the following resources were developed as part of the intervention:

**Table 3:** Evidence base for No CAUTI Bundle

N	NEED for catheter assessed – refer to indications, scan bladder, consider alternative, document indication. - Need for IUC is assessed - appropriate indications for insertion [7, 47, 48]. - Scan the bladder to determine bladder volume [7] - Consider alternatives such as external sheath (males),intermittent catheterisation by staff/patient, SPC, double voiding, commode, timed toileting [7, 47, 48]
O	OBTAIN patient consent, OFFER patient education including hygiene. - Obtain patient consent and importance of accurate complete documentation. - Provide written and verbal information to patient/carer [49] - Ensure daily meatal hygiene is performed as part of personal hygiene, soap and water is all that is required [7, 47, 48]
C	COMPETENCY – clinicians who insert catheters must have documented competency - Competent and trained staff should insert catheters [7, 48]
A	ASEPSIS – maintain asepsis & hand hygiene during insertion and while catheter is in place. - Aseptic technique and sterile equipment must be used for IUC insertion. Hand hygiene “Moment 2” and non-sterile gloves is recommended when manipulation of the IUC or drainage system is required. - Empty the bag when ¾ full and use a clean container for each patient; avoid contact between outlet and container. - Maintain a sterile closed system of drainage [7, 48]
U	UNOBSTRUCTED flow – no kinks or loops, catheter secured, bag below bladder level and off the floor. - Unobstructed continuous urine flow with no kinks or loops, bag below the bladder and not in touch with any surface. Secure the catheter to the patient to minimise movement and trauma and improve patient comfort [7, 48]
T	TIMELY catheter removal and documentation – may be nurse initiated. - Timely removal of the IUC - daily review. Nurse initiated removal guidelines followed if there is no medical documentation for continued use [7, 48]
I	INFECTION risk – daily periurethral hygiene. Collect urine specimen only when clinically indicated. - Infection and catheter specimen urine (CSU) collection: must be collected using aseptic technique, from a newly inserted catheter and before the commencement of antimicrobials - CSU should only be collected if clinically indicated [7, 47, 48]

- IDC insertion criteria guidelines
- Indications for IDC specimen collection
- Nurse-led IDC removal guidelines (Additional file 1)
- Educational resources and compliance auditing tools

The distribution and standardised use of a cost-effective, generic IDC insertion pack forms part of the intervention. The insertion pack includes all equipment required for catheterisation, documentation stickers, and securing devices.

Routine assessment of clinician competency in urinary catheter insertion will be introduced as part of the multifaceted intervention.

### No CAUTI Bundle

The “bundled intervention” framework used in this project is defined as a collection of a number of evidence based practices or steps, vital to achieving improvement in clinical outcomes [39]. The “No CAUTI” bundle was developed during the pilot project, and is based on evidence-based recommendations. The evidence for the bundled intervention is presented in Table 3.

### Implementation strategies

**Fig. 2:**
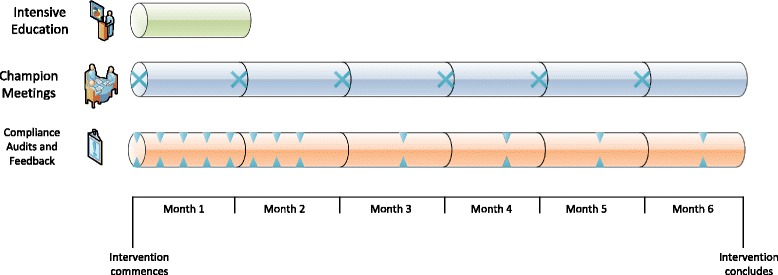
Timeline of implementation components. The intervention commences with four weeks of intensive education. For the first two months, compliance audits are completed on a weekly basis, and then continue on a monthly basis for the remainder of the 6-month intervention period. Champion meetings will be held on a monthly basis throughout the intervention period

A number of implementation strategies will be used in the project: education, monitoring and feedback, resources, and facilitation. The timing of implementation is displayed in Fig. 2. The Template for Intervention Description and Replication (TIDieR) was developed to improve the quality of descriptions of interventions [40], and can be used to report content of behavior change interventions, including what is delivered, who the intervention is delivered to, and what materials are used. TIDieR has previously been used to describe care bundle interventions [41]. The TIDieR framework has been used to outline the current implementation strategies in Table 4.

**Table 4:** “No CAUTI” Implementation strategies summary based on TIDieR

Implementation Strategy	Rationale	Mode of delivery	Delivered by	Delivered to and where	When/how often
Education					
Train-the-trainer workshops	To prepare educators to present the “No CAUTI” bundle to ward-based staff, and to train educators to complete urinary catheterisation competency assessments	Face-to-face (group)	Clinical nurse consultant – urology	Nurse educators from across hospital	1x 2-3 h workshop at each facility at start of intervention
Ward in-services	To familiarise staff with “No CAUTI” bundle and nurse-initiated removal flowchart To identify champions in each ward	Face-to-face (group)	Nurse educators	Nurses and medical officers from all adult inpatient wards, OTs, and EDs	Minimum 1x 20 min in-service in each ward at start of intervention
Monitoring and feedback					
Compliance audits and feedback	To monitor compliance with “No CAUTI” bundle and provide strategies to support implementation	Individual patient audit, and feedback face-to-face (group) to clinicians	Champions (clinicians previously identified in in-services)	All inpatient wards	Weekly for first two months and then monthly for remaining 4 months of intervention period.
Feedback of point prevalence of IDC usage and CAUTI	To focus clinicians on targets and progress	Face-to-face (group) and email	Research project staff	All clinicians at a ward, facility, and district level	Baseline, 4 months, and 9 months
Resources					
“No CAUTI” bundle posters	Prompt awareness and better documentation	Documents displayed in wards	N/A (passive component)	Nurses and medical officers	Ongoing
“No CAUTI” bundle badges	Prompt awareness of intervention and identify ward champions	Worn by clinicians and champions	N/A (passive component)	Nurses and medical officers	Ongoing
Catheter insertion DVDs	Educate nurses about correct catheterisation processes	Available on intranet	N/A (passive component)	Nurses	Ongoing
Facilitation					
Competency assessments	Increase proportion of clinicians that are competent in urinary catheterisation	Face-to-face (individual)	Nurse educators	Nurses	Ongoing
Champions	Act as a resource for clinicians and promote the No CAUTI bundle to clinicians; support implementation	Face-to-face (individual and group)	Nurses	Nurses and medical officers	Ongoing

Whilst there are key implementation strategies that will be common to all intervention hospitals, there will be a degree of flexibility between the two Health Districts, and their hospitals. Both active (e.g. workshops, audit and feedback) and passive strategies (e.g. distribution and display of posters, equipment) will be used [28].

Previous studies have identified champions as playing a significant role in reinforcing practice change [42]. The need for multiple champions when implementing a large degree of practice change is recommended [43]; the current study will have a champion in each ward, and champions will meet regularly. Nursing staff are critical to the success of bundled interventions aimed at reducing IDC use [27].

### Power and sample size calculation

A sample size calculation has indicated that 500 patients per Health District would be sufficient to detect a 40% fall (15 to 9%) in relative IDC insertion rates with a power of 0.8 and alpha 0.05. This is based on a 50% (39.5 to 14.6%) reduction observed in the pilot study [34]. Estimated bed numbers of 860 in Health District 2 and 610 in Health District 1 should thus be more than adequate to provide sufficient power to detect a significant change. Further power will be obtained through having baseline control data and from stratifying the analysis by hospital wards.

### Statistical analysis – point prevalence data

Statistical analysis will be undertaken to determine differences in the prevalence of IDCs between Health District 1 (post) and Health District 2 (no intervention). A mixed methods analysis will compare pre and post data within the groups, across the time frame. Within group data will be stratified according to wards to allow for variation in the case mix of patients between wards. If differences in patient demographics are detected at baseline these will be controlled for in the between group analyses. Data linkage will be used to determine LoS and CAUTI rates for patients at each time point.

### Qualitative analysis

All focus group interviews will be digitally audio-recorded and later transcribed verbatim by a professional transcriber/research assistant. Data will be analysed, coded and themed to low-level themes [44]. Cross-checking of coding will occur within the research team, and emerging themes will be shared within the whole research team as a check on credibility.

Using a mixed methods approach, the quantitative data from the point prevalence survey and the clinician survey will be analysed to inform the questions for the focus groups.

### Economic evaluation

The economic evaluation will be based on a cost-effectiveness analysis to determine whether the multifaceted care intervention is more cost-effective than usual care in reducing CAUTI amongst hospital inpatients. A healthcare provider perspective will be adopted. International guidelines for conducting economic evaluations, as recommended by Drummond et al.[45], and Husereau et al. [46] will be followed. Resource use will be identified using a short data collection instrument. Cost related data collected from usual care and intervention arms will include: materials used for catheterisation, proportion of patients receiving IDC, CAUTI rates, LoS for patients diagnosed with CAUTI, and CAUTI treatment expenses (e.g. antibiotics).

The measure of effect will be based on the change in the rate of CAUTI between the usual care and intervention groups. If the expected intervention benefit is demonstrated in the trial, the measure of effect will be the cases of CAUTI avoided due to the intervention. The economic analysis will identify the cost to avoid an additional case of CAUTI. The reportable outcomes will be average cost-effectiveness and incremental cost-effectiveness ratios. A sensitivity analysis will be conducted to explore the robustness of the results to the uncertainty around parameters used in the model. The results will be interpreted in a broader decision making framework that includes acceptability and sustainability of the intervention. The economic sustainability of the intervention will be based on the cost and effect of delivering the intervention in a wider setting. The analysis will also report the resources required to implement the intervention in other localities. This information is relevant to policy makers because it reflects the resources required by other Health Districts to implement the intervention.

## Discussion

A review of the literature highlighted a lack of interventional studies aiming to reduce IDC use or CAUTI rates in the Australian context. Internationally, there is a sparsity of studies using a control design in CAUTI intervention evaluations.

This study will add to the evidence-base through enhancing understanding of interventions to reduce CAUTI, using a control design to reduce secular effects.

Using the TIDieR framework, implementation strategies have been explicitly outlined, enabling easier replication of the intervention and implementation strategies. The use of a mixed methods approach will provide a platform to explore in-depth the existing barriers and enablers related to implementing practice change.

Ultimately, this study will improve patient safety through implementation and a robust evaluation of clinical practice and practice change.

## Additional file

Additional file 1: Nurse-initiated IDC assessment and removal decision flowchart. (DOCX 152 kb)

## Abbreviations

AHRQ: Agency for Healthcare Research and Quality
CAUTI: Catheter-associated urinary tract infection
CEC: Clinical Excellence Commission
CUSP: Comprehensive Unit-based Safety Program
HAI: Healthcare-associated infection
IDC: Indwelling urinary catheter
LoS: Length of stay
TIDieR: Template for Intervention Description and Replication
UTI: Urinary tract infection.
