# Supplementary material for: Avoiding inappropriate urinary catheter use and catheter-associated urinary tract infection (CAUTI): a pre-post control intervention study
Source: BMC Health Serv Res. 2017 May 2;17:314. doi: 10.1186/s12913-017-2268-2 (PMC5414128; doi:10.1186/s12913-017-2268-2)
Supplement: Additional file 1: — Nurse-initiated IDC assessment and removal decision flowchart. (DOCX 152 kb) [file 12913_2017_2268_MOESM1_ESM.docx]

Additional file 1 modified from Giles et al. 2015 (with author permission) originally published by Healthcare Infection.
